# Optic neuropathy caused by orbital Kimura disease: A rare case report

**DOI:** 10.1097/MD.0000000000030750

**Published:** 2022-09-16

**Authors:** Yung-En Tsai, Yi-Hao Chen, Tung Liu, Ke-Hung Chien, Chih-Kang Hsu

**Affiliations:** a Department of Ophthalmology, Kaohsiung Armed Forces General Hospital, Kaohsiung, Taiwan; b Department of Ophthalmology, Tri-Service General Hospital, National Defense Medical Center, Taipei, Taiwan; c Department of Pathology, Tri-Service General Hospital, National Defense Medical Center, Taipei, Taiwan; d Department of Ophthalmology, Tri-Service General Hospital Songshan branch, Taipe, Taiwan.

**Keywords:** elevated IgE level, Kimura disease, optic neuropathy, orbital involvement, peripheral eosinophilia

## Abstract

Kimura disease (KD) is a rare, chronic inflammatory disease characterized by painless subcutaneous nodules predominantly located in the head and neck regions. Orbital KD, which intrudes into the intraconal space and results in compressive optic neuropathy, is rare and has not been previously reported.

**Patient concerns:**

A 68-year-old man presented with blurred vision and progressive proptosis in the left eye that had been present for 2 years.

**Diagnosis:**

Magnetic resonance imaging of the brain revealed soft tissue lesions with contrast enhancement and restricted diffusion involving the bilateral eyelids, orbits, and intraconal region; those on the left side were more prominent than those on the right side. The lesion encased the left optic nerve. Laboratory test results revealed elevated serum immunoglobulin E level and peripheral eosinophilia. An orbital mass biopsy demonstrated hyperplastic lymphoid follicles with germinal centers in the subcutaneous area and abundant mononuclear and binuclear eosinophils infiltrating the interfollicular area. A pathological diagnosis of KD was made based on the blood test results.

**Interventions:**

Orbital decompression and debulking surgery of the orbital tumor in the left eye were performed to treat the compressive optic neuropathy.

**Outcomes:**

After systemic oral steroid and immunosuppressive agent therapies, the patient’s visual acuity in the left eye improved, and the KD activity was stable.

**Conclusions:**

We present a rare case of orbital KD-associated optic neuropathy, wherein early diagnosis and treatment preserved the patient’s vision. This complication should be considered in patients with a history of compressive optic neuropathy during the differential diagnosis.

## 1. Introduction

Kimura disease (KD) is a rare, chronic inflammatory disease characterized by painless subcutaneous nodules predominantly located in the head and neck regions. KD is primarily observed in young men of Asian descent. Elevated serum immunoglobulin E (IgE) levels and peripheral eosinophilia are common in patients with KD. The masses most commonly occur in the parotid, submandibular, and peri-auricular regions,^[1]^ and orbital involvement is rare. To the best of our knowledge there are no reports of orbital KD that intruded into the intraconal space and resulted in compressive optic neuropathy in the literature available in English. Here, we report a rare case of optic neuropathy resulting from KD.

## 2. Case description

A 68-year-old man presented with blurred vision and progressive proptosis in the left eye that had been present for 2 years. He had a history of KD which had affected the right upper eyelid and had undergone treatment by excision after which systemic steroid therapy was administered 6 years ago. However, the patient was lost to follow-up.

**Figure 1.**
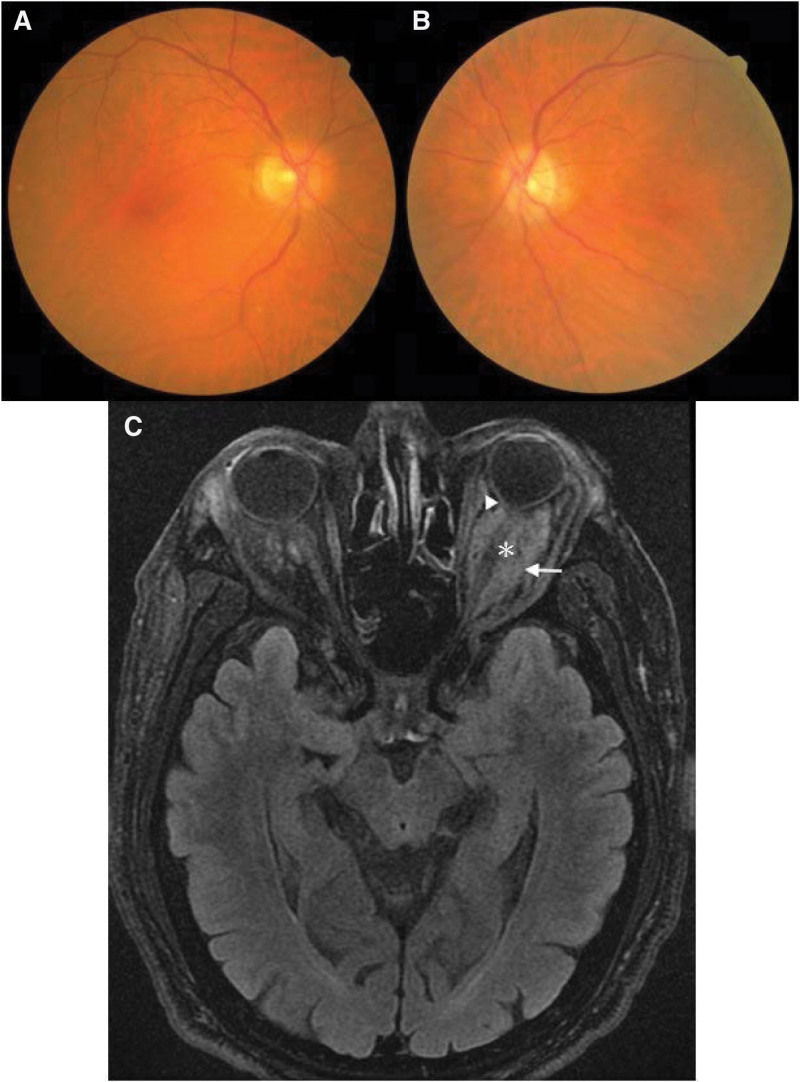
Funduscopic examination showing negative findings in the right eye (A) and vascular tortuosity in the left eye (B). (C) Magnetic resonance imaging of the brain with axial T2 fluid-attenuated inversion recovery demonstrating soft tissue lesions with contrast enhancement and restricted diffusion involving bilateral eyelids, orbits, and intraconal region, which are more prominent on the left side (white arrow) than on the right side. The left optic nerve is encased by lesions (white asterisk). Staphyloma of the left globe is also observed (white arrowhead).

**Figure 2.**
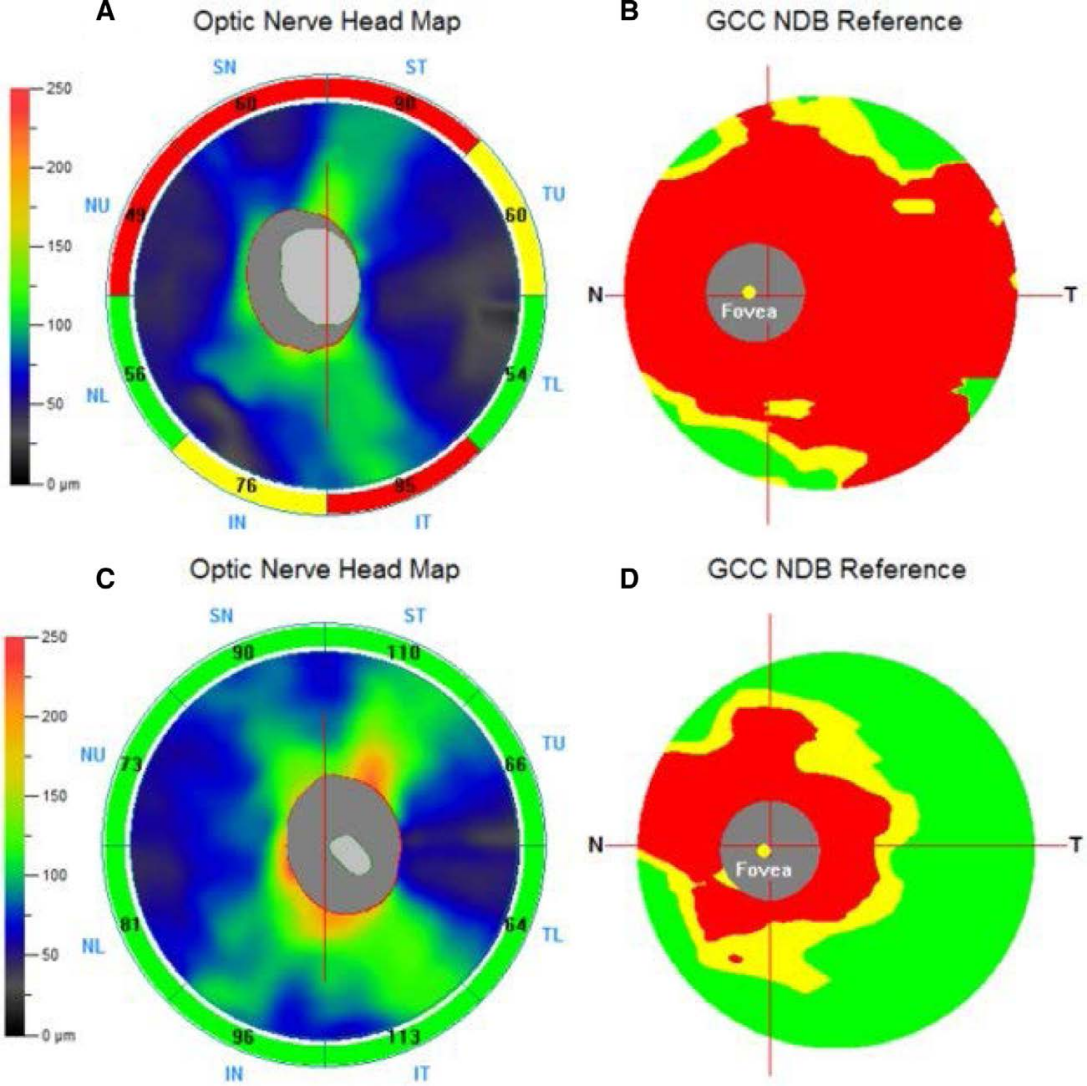
Spectral-domain optical coherence tomography shows the optic nerve head and retinal nerve fiber layer sector average thickness maps of the left eye demonstrating thinning of the nasal-upper, superior-nasal, superior-temporal, and inferior-temporal parts (A). The ganglion cell complex thickness map of the left eye show nearly diffuse thinning (B). After 6 months of treatment, spectral-domain optical coherence tomography shows the optic nerve head and retinal nerve fiber layer sector average thickness maps of the left eye demonstrating in the normal range (C). The ganglion cell complex thickness map of the left eye shows thinning over the peri-foveal region (D); compared with (B), there is a reduction in the thinning area.

Ophthalmologic examination revealed intraocular pressures of 9 mmHg in the right eye and 11 mmHg in the left eye. The left eye demonstrated proptosis and restricted movement in all directions, whereas the right eye movements were intact. The visual acuity of the right eye was 20/25 and that of the left eye was counting fingers at 1 meter. The relative afferent pupillary defect was positive in the left eye. Slit-lamp examination revealed mild bilateral cataracts. Funduscopic examination showed negative findings in the right eye (Fig. 1A) and vascular tortuosity in the left eye (Fig. 1B). Spectral-domain optical coherence tomography (SD-OCT) demonstrated normal in the right eye and nearly diffuse ganglion cell complex (GCC) thinning in the left eye (Fig. 2B). There was thinning of the nasal-upper, superior-nasal, superior-temporal, and inferior-temporal parts of the retinal nerve fiber layer (RNFL) of the left eye (Fig. 2A). The visual field test demonstrated normal of the right eye and nearly complete field loss of the left eye. Magnetic resonance imaging of the brain revealed soft tissue lesions with contrast enhancement and restricted diffusion involving the bilateral eyelids, orbits, and intraconal region, which were more prominent on the left side than on the right side. The lesions encased the left optic nerve. Furthermore, staphyloma of the left globe was observed (Fig. 1C).

**Figure 3.**
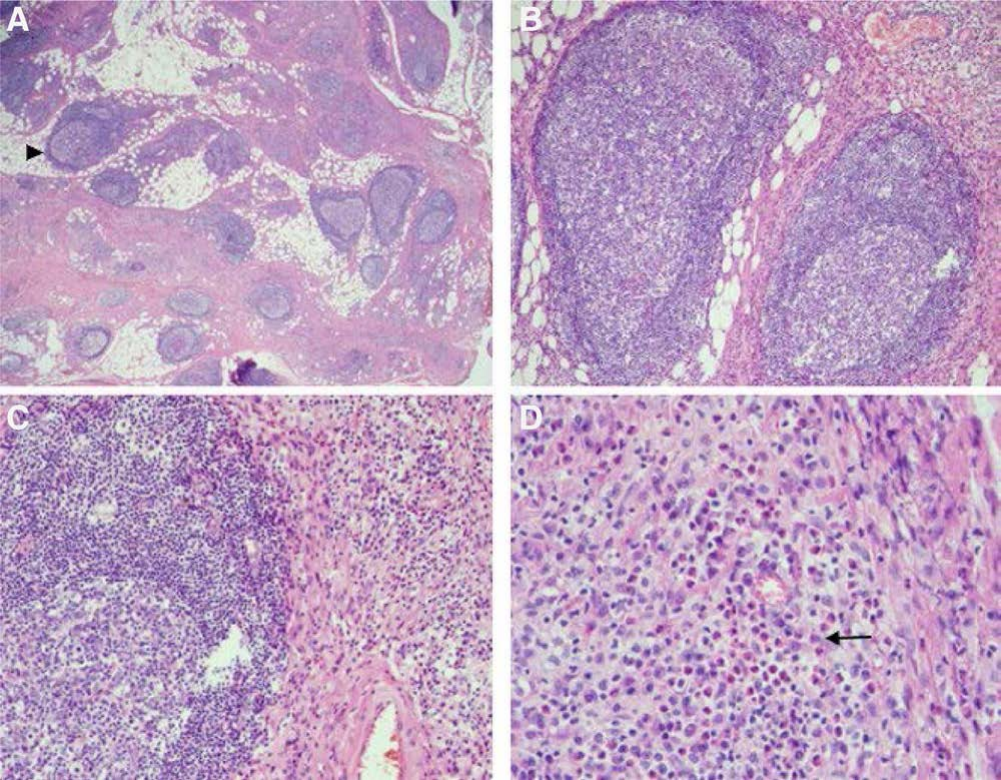
Histologic features of the orbital mass. Hematoxylin and eosin staining shows hyperplastic lymphoid follicles with germinal centers (black arrowhead), an increase in vessels and inflammatory infiltrate within and around the lymphoid follicles (A, 20×; B, 100×; C, 200×), and abundant mononuclear and binuclear eosinophils (black arrow) infiltrating the interfollicular area (D, 400×).

Laboratory examination revealed elevated serum IgE level (1199 IU/mL; normal range, 1.5–144 IU/mL), serum immunoglobulin G4 level (1040 mg/dL; normal range, 5–117 mg/dL), and peripheral blood eosinophil count (1429/μL; 21.2% of the total number of white blood cells; normal range, 0%–6%). Orbital decompression and debulking surgery of the orbital tumor in the left eye were performed to treat compressive optic neuropathy. Biopsy of the orbital mass revealed hyperplastic lymphoid follicles with germinal centers in the subcutaneous area and abundant mononuclear and binuclear eosinophils infiltrating the interfollicular area (Fig. 3).

Based on the blood test results, a pathological diagnosis of KD was made. The patient was started on oral corticosteroids, and long-term oral administration of cyclophosphamide (50 mg/week) was initiated to modulate immunity. There was reduction in proptosis and improvement of movement in all directions in the left eye. After 6 months of treatment, the visual acuity of the left eye improved from counting fingers at 1 meter to 20/63, and SD-OCT demonstrated improvement in the GCC (Fig. 2D) and RNFL (Fig. 2C) thinning of the left eye.

## 3. Conclusions

KD is a benign, chronic granulomatous inflammatory disorder of unknown etiology, although it is hypothesized to represent an allergic or autoimmune process.^[2]^ In 1937, this condition was first described by Kimm and Szeto in 7 cases from China.^[3]^ The disease was officially designated by its current name in 1948 because Kimura et al. characterized the pathological features of the disease.^[4]^

The epidemiology of KD revealed that while young Asian men are the most commonly affected, it can occur in both men and women belonging to different ethnicities and age groups. The typical triad of clinical symptoms include unilateral painless cervical adenopathy or subcutaneous masses largely in the head and neck region, characteristic histologic features, as well as peripheral eosinophilia, and elevated serum IgE levels.^[5]^ Histologically, KD is identified by follicular hyperplasia with active germinal centers along with a proliferation of arteriolar and capillary vasculature encompassed by concentric rings of reactive fibrosis composed of hyaline and collagen. Numerous lymphocytes and prominent eosinophils infiltrate the interfollicular zones.^[6]^

Takenaka et al reported the first case of orbital KD in 1976.^[7]^ It usually affects the lacrimal glands or eyelids, although its occurrence is infrequent.^[8]^ Furthermore, there are no known cases of optic neuropathy caused by KD. Optic neuropathy is caused by damage to the optic nerve that may occur because of several reasons such as ischemia, compression, inflammation, infection, hereditary conditions, toxins, and trauma. In cases of compressive optic neuropathy, lesions within the orbit caused by tumors, infections, and inflammation can compress the optic nerve, resulting in progressive visual loss. Compressive optic neuropathy also causes thinning of the GCC and RNFL which can be observed on SD-OCT scans^[9]^ and may be confirmed or ruled out by magnetic resonance imaging of the brain and orbits. While there is currently no definitive, optimal treatment for KD, surgical excision of the orbital or adnexal lesions is recommended. Complete excision may be difficult because of the unclear margins of these lesions. However, if excision is incomplete, recurrence is common. Although the side effects of radiotherapy are considerable, it has been used successfully in a few cases.^[10]^ KD generally responds well to oral or intravenous steroids, although it often recurs after steroid withdrawal. If patients are refractory to steroid treatment, they may be treated using systemic immunosuppressive agents.^[2]^

We described a rare case of optic neuropathy associated with orbital KD. Our patient was treated using orbital decompression along with debulking surgery of the orbital tumor. Subsequently, systemic oral steroids and immunosuppressive agents were administered. After 6 months of treatment, the thickness of the GCC and RNFL as well as visual acuity improved. Early diagnosis and treatment aided in the preservation of the patient’s vision. KD should be considered as part of the differential diagnosis in patients with mass-induced compressive optic neuropathy when accompanied by peripheral eosinophilia and elevated serum IgE levels.

## Acknowledgments

The authors would like to thank the patient for his participation in this study.

## Author contributions

Conceptualization: Chih-Kang Hsu.

Data curation: Ke-Hung Chien, Chih-Kang Hsu.

Methodology: Yi-Hao Chen, Chih-Kang Hsu.

The histological and pathological examination: Tung Liu.

Writing – original draft: Yung-En Tsai.

Writing – review & editing: Chih-Kang Hsu.

All authors read and approved the final manuscript.
